# Patient factors associated with the use of psychotropic polypharmacy in patients under the care of a community mental health team in the West of Ireland

**DOI:** 10.1192/bjo.2021.314

**Published:** 2021-06-18

**Authors:** Karthika Srikumar, Richard Walsh, Donnchadh Walsh, Sonn Patel, Sheila O'Sullivan

**Affiliations:** 1 Acute Adult Mental Health Unit; 2 School of Medicine, University College Dublin

## Abstract

**Aims:**

Psychiatric polypharmacy refers to the prescription of two or more psychotropic medications to any one patient. This definition is purely quantitative and does not take into account whether such a prescription is detrimental, or unnecessary. In many cases, polypharmacy has been implemented in challenging illnesses, and some studies have shown that it can improve overall outcomes for certain patients. Evidence suggests that the prevalence of psychotropic polypharmacy is increasing, despite advances in psychosocial interventions. The aim of this study was to assess the current prevalence of polypharmacy among patients being treated by a community mental health team (CMHT), and the patient factors associated with its use.

**Method:**

We performed a cross-sectional study of all patients registered with a CMHT in a mixed urban/rural area on a single date. Case records were examined to determine the most recently prescribed drug regimen for each patient. Clinical chart diagnoses were recorded and each one independently verified by the team consultant using ICD-10. A number other sociodemographic variables were recorded. Using Microsoft Excel, we analysed the medications prescribed as well as rates and levels of polypharmacy based on multiple different patient characteristics.

**Result:**

Of the 245 patients, the mean age was 56.3 and 51.2% (n = 126) were female. Psychotropic polypharmacy was seen in 62% (n = 152) of patients. 33% (n = 82) of patients were on two psychotropic medications, and of this subset, a combination of one antipsychotic and one antidepressant was the most common drug regimen, seen in 16.7% (n = 41) of all patients. Polypharmacy was more prevalent in females, with 68% (n = 85) being on two or more psychotropics, in comparison to 58% of male patients. In relation to age, patients aged between 51 to 65 years had the highest prevalence of polypharmacy, at a rate of 71% (n = 49). Among all primary diagnoses, polypharmacy was most common in patients with affective disorders, with 80% (n = 40) of this patient cohort on two or more medications. Second to this was psychotic disorders, with polypharmacy seen in 65% (n = 62) of this group.

**Conclusion:**

We found that psychotropic polypharmacy is highly prevalent in psychiatric patients being treated in a community setting. Certain demographics and patient factors, such as age, gender and psychiatric diagnosis influenced the rate of polypharmacy and certain drug combinations were more commonly prescribed than others.

